# The impact of tumor profiling approaches and genomic data strategies for cancer precision medicine

**DOI:** 10.1186/s13073-016-0333-9

**Published:** 2016-07-26

**Authors:** Andrea Garofalo, Lynette Sholl, Brendan Reardon, Amaro Taylor-Weiner, Ali Amin-Mansour, Diana Miao, David Liu, Nelly Oliver, Laura MacConaill, Matthew Ducar, Vanesa Rojas-Rudilla, Marios Giannakis, Arezou Ghazani, Stacy Gray, Pasi Janne, Judy Garber, Steve Joffe, Neal Lindeman, Nikhil Wagle, Levi A. Garraway, Eliezer M. Van Allen

**Affiliations:** 1 Department of Medical Oncology, Dana-Farber Cancer Institute, Harvard Medical School, 450 Brookline Avenue, Boston, MA 02115 USA; 2 Broad Institute of MIT and Harvard, 7 Cambridge Center, Cambridge, MA 02142 USA; 3 Department of Pathology, Brigham and Women’s Hospital, Boston, MA USA; 4 Department of Medical Ethics and Health Policy, University of Pennsylvania, Philadelphia, PA USA; 5 Center for Cancer Precision Medicine, Dana-Farber Cancer Institute, 450 Brookline Avenue, Boston, MA 02115 USA

**Keywords:** Genomics, Precision medicine, Disparities, Immuno-oncology, Neoantigens, Panel testing

## Abstract

**Background:**

The diversity of clinical tumor profiling approaches (small panels to whole exomes with matched or unmatched germline analysis) may engender uncertainty about their benefits and liabilities, particularly in light of reported germline false positives in tumor-only profiling and use of global mutational and/or neoantigen data. The goal of this study was to determine the impact of genomic analysis strategies on error rates and data interpretation across contexts and ancestries.

**Methods:**

We modeled common tumor profiling modalities—large (*n* = 300 genes), medium (n = 48 genes), and small (*n* = 15 genes) panels—using clinical whole exomes (WES) from 157 patients with lung or colon adenocarcinoma. We created a tumor-only analysis algorithm to assess germline false positive rates, the impact of patient ancestry on tumor-only results, and neoantigen detection.

**Results:**

After optimizing a germline filtering strategy, the germline false positive rate with tumor-only large panel sequencing was 14 % (144/1012 variants). For patients whose tumor-only results underwent molecular pathologist review (*n* = 91), 50/54 (93 %) false positives were correctly interpreted as uncertain variants. Increased germline false positives were observed in tumor-only sequencing of non-European compared with European ancestry patients (*p* < 0.001; Fisher’s exact) when basic germline filtering approaches were used; however, the ExAC database (60,706 germline exomes) mitigated this disparity (*p* = 0.53). Matched and unmatched large panel mutational load correlated with WES mutational load (r^2^ = 0.99 and 0.93, respectively; *p* < 0.001). Neoantigen load also correlated (r^2^ = 0.80; *p* < 0.001), though WES identified a broader spectrum of neoantigens. Small panels did not predict mutational or neoantigen load.

**Conclusions:**

Large tumor-only targeted panels are sufficient for most somatic variant identification and mutational load prediction if paired with expanded germline analysis strategies and molecular pathologist review. Paired germline sequencing reduced overall false positive mutation calls and WES provided the most neoantigens. Without patient-matched germline data, large germline databases are needed to minimize false positive mutation calling and mitigate ethnic disparities.

**Electronic supplementary material:**

The online version of this article (doi:10.1186/s13073-016-0333-9) contains supplementary material, which is available to authorized users.

## Background

The mapping of the human genome, together with the advent of massively parallel sequencing, has accelerated discovery of driver genetic alterations in cancer and the development of drugs to target or otherwise exploit these events [1]. Multiple tumor profiling approaches that leverage these advances have entered the clinic. Such assays often consist of targeted sequencing panels that query a subset of typically 200–500 genes implicated in cancer biology or clinical management [2–8]. Alternatively, panels that emphasize rapid turnaround time by profiling smaller gene sets (n = 15–48 genes) have also emerged [9, 10]. On the other end of the spectrum, clinical whole-exome sequencing (WES; n ~ 20,000 genes) of matched tumor and germline samples has been studied through prospective sequencing efforts [11–13]. However, the benefits and limitations of these different sequencing strategies remain incompletely understood.

Understanding the differences in genomic results between different tumor profiling approaches will become increasingly important as the cancer genome is leveraged to stratify patients for new therapeutic strategies. For example, unlike targeted therapies linked to specific genetic lesions (e.g., epidermal growth factor receptor mutations and inhibitors), immune targeting strategies, such as checkpoint blockade or personalized cancer vaccines, may require large-scale ascertainment of mutational and neoantigen loads and individual mutation-associated neoantigens for personalized cancer vaccine development [14–18]. One effort demonstrated the ability of two large gene panels (315 or 573 genes) to predict mutational load for immunotherapy response in pilot patient cohorts [19], and another effort demonstrated the ability of one large gene panel (341 genes) to predict DNA mismatch repair protein deficient tumors through mutational load [20], although a systematic characterization of different tumor profiling strategies for both mutation load and personal neoantigen identification should inform their relative utilities for stratifying patients in emerging cancer precision medicine frameworks.

Moreover, although sequencing of paired normal blood or tissue samples is standard practice for research-oriented WES applications, many targeted panel approaches do not include matched normal samples [2, 3, 9, 21, 22]. Together with the limited ancestral diversity in many existing germline databases, this absence of paired normals has raised concerns for the potential of increased false positive somatic mutation calls that are actually germline [23, 24].

To investigate these issues, we analyzed clinical sequencing data from 157 patients with advanced lung and colon adenocarcinoma to ascertain the relative merits of distinct tumor profiling approaches.

## Methods

### Patients and tumor specimens

All patients consented to an institutional review board-approved protocol that allows comprehensive genetic analysis of tumor and germline samples (Dana-Farber Cancer Institute #12-078). Ancestry status was self-reported. Samples were selected from pathology archives by a board-certified anatomic pathologist based on sample size, tumor purity, and timing relative to date of study enrollment and analyzed by the Center for Advanced Molecular Diagnostics (CAMD) at Brigham and Women’s Hospital (BWH), a Clinical Laboratory Improvements Amendments (CLIA)-certified laboratory. Tumor content was estimated by an anatomic pathologist from corresponding stained slides and only samples with at least 20 % malignant cells were analyzed. DNA was isolated with a commercial kit (QIAamp DNA Mini Kit, Qiagen, Valencia, CA, USA) following the manufacturer’s instructions. DNA was quantified (PicoGreen, ThermoFisher Scientific, Waltham, MA, USA) and samples with at least 50 ng/μL of DNA proceeded to library preparation.

### Whole exome sequencing

WES from formalin-fixed, paraffin embedded (FFPE) samples was performed as described previously [12]. Whole-exome capture libraries were constructed from tumor and normal DNA after sample shearing, end repair, phosphorylation, and ligation to barcoded sequencing adaptors. DNA was then subjected to solution-phase hybrid capture using Agilent baits. The samples were multiplexed and sequenced using Illumina HiSeq technology. All BAM files were deposited in dbGap phs001075.v1.p1.

### Genomic analysis

#### Sequence data processing and quality control

WES data were processed using established analytical pipelines at the Broad Institute [12]. A BAM file was produced using the Picard pipeline (http://broadinstitute.github.io/picard/), which aligns tumor and normal sequences to the hg19 human genome build from raw Illumina reads using the BWA aligner (version 0.5.9-tpx [0.5.9 with an internal patch to support threading]). BAM files were uploaded into the Firehose pipeline (http://www.broadinstitute.org/cancer/cga/Firehose), which manages input and output files to be executed by GenePattern [25]. Quality control modules within Firehose were applied to all sequencing data for comparison of the origin for tumor and normal genotypes and to assess fingerprinting concordance. Cross-contamination of samples was estimated using ContEst [26]; those with >5 % contamination were excluded from subsequent analysis.

#### Somatic alterations and downsampling

MuTect (version 1.1.6) [27] was applied to identify somatic single-nucleotide variants. Artifacts introduced by DNA oxidation during sequencing or from FFPE were computationally removed using a filter-based method [28]. Annotation of identified variants was done using Oncotator (version 1.2.7.0) [29]. Representative large (*n* = 300) [3], medium (*n* = 48), and small (*n* = 15) [9] gene sets were defined through review of literature (Additional file 1: Table S1; Additional file 2: Table S2; Additional file 3: Table S3). The aggregate mutation data across the whole exome were collected in two files, one containing all tumor-germline matched calls (Additional file 4: Table S4) and a second containing tumor-only calls (Additional file 5: Table S5). “Downsampling” was performed on the aggregate somatic mutation alteration data files to derive subsets of WES data for gene sets represented by the large, medium, and small gene lists defined by the respective panels. For example, to model the 300 gene panel, the set of mutations from the entire WES data was restricted to only consider events in those 300 genes. The analyses were performed using the R statistical software.

#### Tumor-only and germline analysis

Tumor-normal paired mutation data were taken to be the set of all true somatic mutations for each patient. To ensure only high-confidence mutation calls were considered, only mutations with an allelic fraction ≥5 % and sequencing or FFPE artifact filtering strategies described above were considered. Tumor-only mutation calling was performed by using MuTect and pairing the tumor WES with an FFPE germline whole exome from another patient to reduce false positive calls introduced by artifacts from the sequencing process, as described previously [23]. Variants were removed if they were present in combinations of dbSNP (build 134) [30] and 1000 Genomes (phase 1, version 3) [31] using the Oncotator annotation algorithm [29], along with the ExAC (version 0.3) [32] databases. Mutations were rescued if listed as somatic in COSMIC (version 74) at least one, three, five, or ten times, for increasing stringency [33]. Positive predictive values for each filter were calculated by dividing the number of true somatic mutations in the post-filtering mutation data by the total number of unfiltered mutations. Sensitivity was calculated by dividing the number of true somatic mutations in the tumor-only post-filtering data by the total number of known somatic mutations in the paired mutation data. To obtain the set of known germline variants, the GATK HaplotypeCaller (version 3.1.1) [34, 35] was applied to germline sequence BAMs to identify germline single-nucleotide polymorphisms (SNPs) using WES data from each patient. Unfiltered germline variants in tumor-only targeted panel data after application of various database filters were identified by comparison with exome germline SNP data.

#### Orthogonal large panel molecular pathology review

A subset of cases (*n* = 91) underwent separate testing with an academic lab large gene panel (“OncoPanel”), followed by molecular pathology review [3]. Specifically, after variants were identified by computational approaches, an individual molecular pathologist reviewed each variant and assigned a tier based on clinical actionability and to determine whether the variant was likely somatic or germline and whether there were any clinical actions for the variant. The four tiers in this system are:

1. The alteration has well-established published evidence confirming clinical utility in this tumor type in at least one of the following contexts: predicting response to treatment with a US Food and Drug Administration (FDA)-approved therapy; assessing prognosis; establishing a definitive diagnosis; or conferring an inherited increased risk of cancer to this patient and family.
2. The alteration may have clinical utility in at least one of the following contexts: selection of an investigational therapy in clinical trials for this cancer type; limited evidence of prognostic association; supportive of a specific diagnosis; proven association of response to treatment with an FDA-approved therapy in a different type of cancer; or similar to a different mutation with a proven association with response to treatment with an FDA-approved therapy in this type of cancer.
3. The alteration is of uncertain clinical utility but may have a role as suggested by at least one of the following: demonstration of association with response to treatment in this cancer type in preclinical studies (e.g., in vitro studies or animal models); alteration in a biochemical pathway that has other known, therapeutically targetable alterations; alteration in a highly conserved region of the protein predicted, in silico, to alter protein function; or selection of an investigational therapy for a different cancer type.
4. The alteration is novel or its significance has not been studied in cancer.

#### Mutation rates and neoantigens

The mutation rate for each sample was calculated by dividing the number of bona fide mutation calls post-filtering by the total genomic territory sequenced (in megabases). Germline exome data from each patient were used to genotype human leukocyte antigen (HLA) loci with POLYSOLVER [36]. Patient HLA genotypes and matched exome mutation data were used as inputs for NetMHCPan [37] to generate predicted binding affinities of somatic mutations linked to specific MHC class I molecules. Predicted mutation-associated neoantigens were defined as all 9- and 10-amino-acid peptides resulting from tumor-specific mutations with predicted HLA binding affinities <500 nM using NetMHCpan (v2.4). Downsampled panel data were queried for exome neoantigens to determine the fraction of putative neoantigens observed in WES that were recoverable from panel mutation calls.

#### Statistical tests

The sample size was based on available material, and thus there was not an a priori power calculation. Comparisons of germline false positive rates between ancestry groups was performed with two-sided Fisher’s Exact test. Pearson correlation tests were performed for mutational and neoantigen load comparisons in the three panel settings.

## Results

### Reducing false-positive germline variants in tumor-only analysis

The study included 157 patients, who underwent clinical sequencing: 75 with colorectal adenocarcinoma and 82 with lung adenocarcinoma. Clinical tumor and germline WES [12] produced a mean coverage of 154× and 133× in tumor and normal DNA, respectively (Additional file 6: Table S6). The combination of sequencing depth and tumor purity (“Methods”) enabled mutation detection in these cases. Large (*n* = 300) [3], medium (*n* = 48), and small (*n* = 15) [9] gene panel data were produced by creating subsets of the whole exome mutation data (“downsampling”) to simultaneously model the different gene sets captured in multiple representative academic and commercial efforts. Matched tumor–germline sequencing revealed a median of 75 (interquartile range of first and third quartiles [IQR] = 55–134), 4 (IQR = 3–6), 2 (IQR = 1–3), and 1 (IQR = 1–2) mutations per patient for WES, large, medium, and small panels, respectively. Unmatched tumor-only sequencing produced a median of 445 (IQR = 404–531), 10 (IQR = 8–13), 3 (IQR = 2–5), and 2 (IQR = 1–2) mutations per patient. Thus, the proportion of putative somatic variants was increased in tumor-only sequencing data under all conditions.

**Fig. 1:**
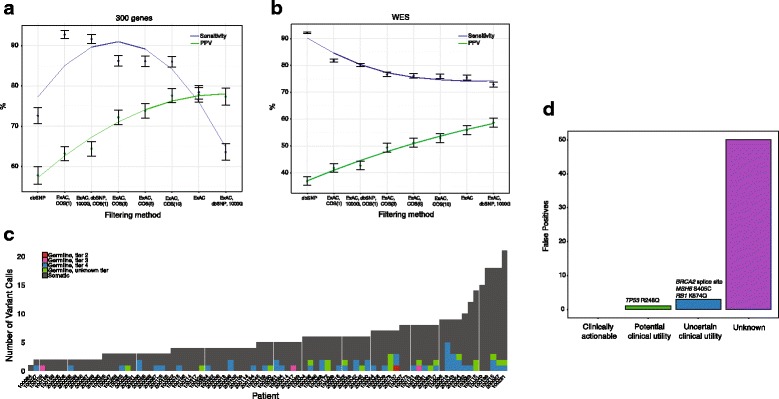
Germline false positives in tumor-only clinical sequencing. Sensitivity and positive predictive value (*PPV*) curves for multiple germline filtering strategies identifies optimal approaches for unmatched large targeted panel testing (**a**) and whole-exome sequencing (**b**). For 91 patients, germline exome data were used to identify false positives post-filtering. Subsequent molecular pathologist review of variants was performed on individual cases to further classify putative germline variants. With molecular pathology review, 50/54 false positive variants were correctly classified as unknown (“tier 4”), with the remaining variants classified as having uncertain (*n =* 3; “tier 3”) or potential (*n* = 1; “tier 2”) clinical utility (**c**, **d**). Please see “Methods” for detailed descriptions of the four-tier classification schema

For all targeted panel options, both sensitivity and positive predictive value (PPV) could be optimized by using an analytical pipeline that consisted of an unmatched germline sample, the largest publically available germline WES database (ExAC) [32], and recovery of somatic mutations with COSMIC at the highest frequency threshold (*n* ≥ 10 events) to recover mutational hotspots [38] (Fig. 1a, b; “Methods”; Additional file 7: Table S7). For large tumor-only targeted panels, this filtering led to 14 % (144/1012 variants) of putative somatic mutations that were actually germline false positive variants (Fig 1c). For unmatched WES, the germline false positive rate with this approach was even higher (18 %; 5282/29,738 variants).

A subset of these cases (*n* = 91) underwent orthogonal molecular pathologist review of variants (“Methods”). The addition of pathologist review after in silico analysis resulted in 50/54 (93 %) false positives interpreted as unknown variants (tier 4) that may be germline false positives rather than somatic alterations (Fig. 1c, d). Of the four remaining germline false positive results, three (*RB1* K874Q, *MSH6* S405C, BRCA2 splice site mutation at the junction of exons 2 and 3 (g.32890665G > A)) were classified as uncertain clinical utility and only one, a known pathogenic variant associated with hereditary cancer syndromes (*TP53* R248Q), was classified as having potential clinical utility based on negative prognostic implications (Fig. 1d).

### The impact of ancestry on germline false positives in tumor-only analysis

**Fig. 2:**
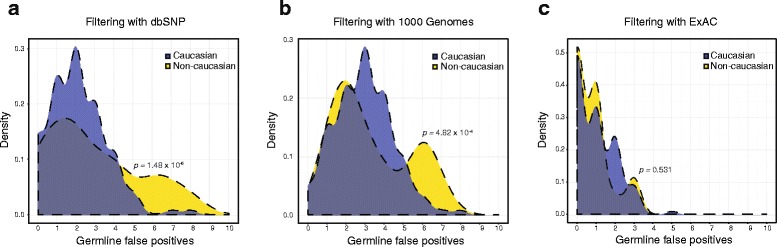
Ancestry and germline false positives using different analysis strategies. **a** The use of dbSNP as the primary germline filtration strategy results in a significant increase in false positives among non-white patients (p < 0.001). **b** A similar increase was observed with the use of 1000 Genomes (*p* < 0.001). **c** With larger germline databases such as ExAC, this disparity is mitigated

The use of germline databases is a critical component for removing false positive germline calls in tumor-only panel sequencing (“Methods”); however, the representation of non-European ancestry in these databases is incomplete [39]. Therefore, we next sought to measure the variation in false positive rates in populations with different self-reported ancestries. When the dbSNP database was used as a filter in large panel tumor profiling analysis [30], germline false positives were significantly increased in tumor-only sequencing of non-European compared with European ancestry patients (odds ratio [OR] = 2.52, *p* < 0.001; Fisher’s exact; Fig. 2a). While the median number of false positives among the two populations was the same (*n* = 2), 32 % (6/19) of non-white patients had more than five false positives, whereas 5 % (7/132) of white patients had more than five germline false positives.. A similar relationship was observed with the use of 1000 Genomes (OR = 1.83, *p* < 0.001; Fisher’s exact; Fig. 2b). However, use of ExAC [32], a public database of 60,706 germline exomes that represents an order-of-magnitude increase in germline variant data compared with other databases, mitigated this disparity (OR = 1.19, *p* = 0.53; Fig. 2c). Therefore, tumor-only mutational profiles require the use of germline databases with sufficiently broad representation to minimize the elevations in false positives that might otherwise be seen in patients with diverse ethnic backgrounds.

### Impact of gene panel size on mutational load and neoantigen prediction

**Fig. 3:**
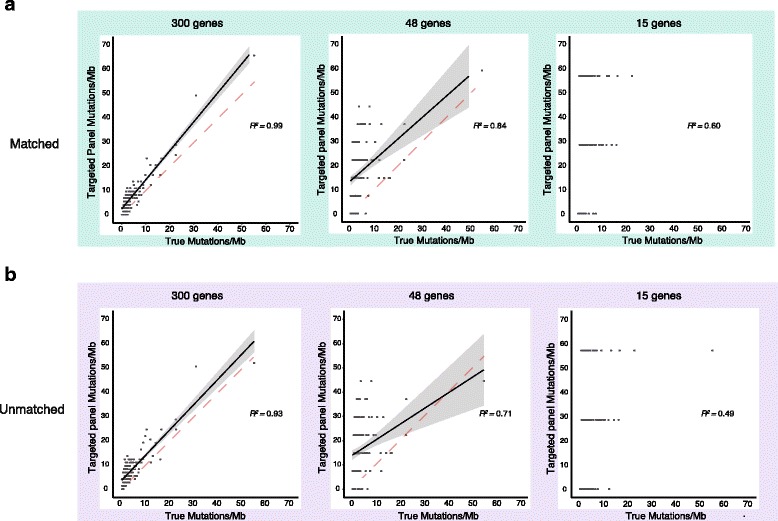
Mutational load predictions with different panel tests. Comparison of mutational load predictions using WES or either matched (**a**) or unmatched (**b**) large panel tests (*n* = 300 genes) demonstrates both can reliably predict the mutational load. The *linear regression line* is shown in *black* with *95 % confidence bands* shaded in *grey*. The *identity line* (*dashed*) is shown for comparison. With medium sized panels (*n* = 48 genes), this ability decreases in both the matched and unmatched setting and is not possible with small (*n* = 15) gene panels

Given the potential utility for immuno-oncology patient stratification, we next investigated the extent to which various tumor profiling platforms might inform genome-wide properties. First, we explored whether mutational loads measured directly (using paired WES data) correlated with mutational loads inferred from targeted panel data (e.g., <2 % of the genomic territory covered by WES). To test this, we divided the number of mutations observed in the panel by the genomic territory covered (in megabases) by that panel (“Methods”). Consistent with previous reports [19], we found that large-panel mutational loads correlated strongly with WES-based mutational load regardless of whether tumor-only or paired data were used (r^2^ = 0.99 for matched and 0.93 for unmatched; *p* < 0.001), with median nonsynonymous mutation rates of 2.3/Mb (IQR = 1.7–4.2) and 5.5/Mb (IQR = 4.1–8.2) in WES and panels, respectively (Fig. 3a, b). The ability of medium gene panels to predict the WES mutational load was somewhat reduced (r^2^ = 0.84 and 0.71, respectively). Small panels were poor predictors of overall mutational load (0.4 < r^2^ ≤ 0.6 for all conditions). When analyzed separately, the lung and colon cancer subsets achieved comparable results across the three settings (Additional file 8: Figure S1; Additional file 9: Figure S2). Thus, large matched or unmatched panels successfully recapitulated the WES mutational load.

**Fig. 4:**
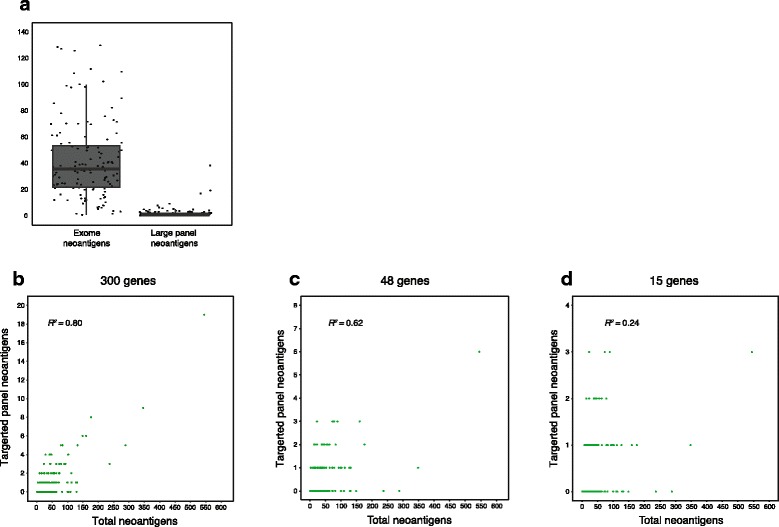
Neoantigen predictions in panels. **a** The proportion of neoantigens called in large panel targeted sequencing data demonstrates an inability to identify as a broad spectrum of neoantigens compared to WES. **b** Nonetheless, there is a linear relationship between large panel neoantigens recovered from exome and germline-matched large panel data. This linear relationship no longer holds when considering neoantigen data from medium (**c**) and small (**d**) targeted panels

Given the potential importance of identifying patient-specific neoantigens—novel protein sequences absent from the normal human genome that arise from somatic mutations [40]—for immuno-oncology applications [16–18, 41], we sought to identify neoantigens derived from the different panels and WES. To do this, we integrated patient human leukocyte antigen (HLA) typing [42] with the set of all potential neoantigens to identify those with predicted high affinities to the patient’s MHC class I alleles (“Methods”). The median neoantigen load across all patients as determined by WES data was 38 neoantigens per exome, while the median number of those called by large panel targeted sequencing was one neoantigen per panel (Fig. 4a). The median number of neoantigens called in both small and medium panels was zero. No correlation was discovered between small/medium panel neoantigen calls and exome neoantigen calls (r^2^ = 0.24 and r^2^ = 0.62, respectively); however, we did observe a correlation between large panel neoantigen and exome neoantigens (r^2^ = 0.81) (Fig. 4b–d). In the WES data, 5511 neoantigens with binding affinities of <500 nM were identified across the 157 patient samples. Of these, 229 (4.1 %) were observed through matched targeted panel sequencing data (Fig. 4a). Thus, while large panels were able to recapitulate mutation and predicted neoantigen loads, most potentially immunogenic neoantigens occurred in genes that were not represented in these cancer panels and would not be observable for cancer vaccine strategies.

## Discussion

As precision medicine efforts proliferate in clinical oncology settings, a spectrum of tumor gene profiling strategies—from individual variant testing to comprehensive WES—are being utilized. We sought to assess the relative merits of each by analyzing genomic data from 157 lung and colon adenocarcinoma cases, followed by in silico modeling of different forms of targeted panel testing to determine their analytical strengths and weaknesses.

One priority was to determine best practices for germline filtering in the setting of tumor-only profiling. While our results identified a consistent (albeit lower) rate of germline false positive findings when leveraging large, publically available germline variant data sets, we found that the addition of molecular pathologist review was highly effective in reducing false positive errors germane to unmatched sequencing. This observation may be informative in centers where assembling a molecular tumor board may not be practically feasible but individual molecular pathologists can act as reviewers.

Even so, the higher false positive germline variants in non-white ancestries highlights the limitations of utilizing germline genomic databases for such filtering, as these cohorts may not represent the clinical population being tested. Tumor-only analytical pipelines that do not anticipate diverse ancestry could unwittingly produce a higher rate of germline false positives in some ancestral backgrounds. Expansion of germline databases to represent the diversity of patients tested is necessary to mitigate this source of false positives, and this strategy may contribute to the improvement of precision medicine health disparities resulting from analytical features of the human genome. Indeed, as clinical genomic profiling becomes increasingly expansive technologically, with whole-genome and whole-transcriptome sequencing being performed in clinical settings, the need to capture a diverse set of patients is especially relevant.

Furthermore, because of the emerging therapeutic avenues associated with tumor neoantigens and mutational load, we sought to analyze the ability of targeted panels to identify patients who might benefit from such treatments. Indeed, targeted large panel mutation rate approximated the WES mutation rate well for most samples, whether the targeted panel was matched or unmatched. This suggests that large targeted panels may be useful for flagging patients with exceptionally high mutation rates for specific clinical investigations. Yet, targeted panel sequencing results failed to recapitulate the neoantigen load estimated from WES data, which may be as relevant as mutational load data when combined with immunohistochemistry markers (i.e., PD-L1 staining) to stratify patients for immunotherapies [43].

In addition, since only a small fraction of total neoantigens fell within the genomic regions covered by the targeted panel (average tumor sample had less than three neoantigens called in the targeted sequencing data), it is likely impossible to stratify patients based on relative neoantigen loads from targeted sequencing data alone. Also, the targeted panel does not call most of the patient-specific neoantigens themselves, which may become increasingly relevant as personalized cancer vaccine strategies requiring knowledge of specific neoantigens expand across many clinical settings [44–47].

The main limitation of this study is that, due to the rapidly expanding diversity of panel-based sequencing approaches offered in commercial and academic labs, it was not possible to directly and comprehensively compare outputs of all available approaches with these clinical samples. As a result, certain components of the workflow could not be examined in this context, such as the impact of higher sequencing depth on variant detection sensitivity [8] or the differences in germline false positive results from different lab analysis processes. Indeed, since not all labs report specific details about analysis methods, such as how exactly germline variants are filtered, we could not confirm whether the approach outlined in this effort is consistent across vendors. This highlights the importance of encouraging transparency in analytical efforts given how widely variable results may seem depending on which approach is used. Since germline variants may also have immediate clinical implications for assigning cancer risk [48] and therapeutic strategies [49], distinguishing somatic and germline events is especially relevant in this context. Furthermore, this study highlights certain benefits of WES, although WES compared with panel testing has additional costs (i.e., financial, interpretive) beyond analytical.

## Conclusions

Broadly, our work highlights the relative advantages and disadvantages of WES and targeted panel sequencing for clinical precision oncology. Targeted panel sequencing maintains an advantage over WES for variant identification in a small set of known clinically informative cancer genes and utilization of germline enhances somatic mutation identification. Additionally, prior studies have demonstrated that targeted panels enable more sequencing depth compared with WES. Since the WES obtained for this study was considered sufficient to enable the subsequent analyses, this technical component of targeted panels did not require further exploration to enable the studies described herein.

Even so, the breadth and adaptability of WES may ultimately offer advantages over targeted panels for certain immunotherapy regimens. As treatment paradigms shift and require detailed assessments of global genomic changes for immunotherapy purposes, as well as deep clonal architecture of tumors feasibly enabled through deeper targeted sequencing, a combination of these strategies may prove most effective for genomic analysis in the clinic. When paired with up-to-date bioinformatics and database filtering, along with molecular pathology assessment, this strategy may inform wider analytical standardization for genomic analysis.

## Additional files

Additional file 1: Table S1. Large panel genes. (DOCX 29 kb) Additional file 2: Table S2. Medium panel genes. (DOCX 20 kb) Additional file 3: Table S3. Small panel genes. (DOCX 19 kb) Additional file 4: Table S4. All matched mutation calls (due to size, see Additional file 5: Table S5 for unmatched mutation calls). (XLSX 3746 kb) Additional file 5: Table S5. All unmatched mutation calls (due to size, due to size, see Additional file 4: Table S4 for matched mutation calls). (XLSX 11186 kb) Additional file 6: Table S6. Sample sequencing metrics. (DOCX 27 kb) Additional file 7: Table S7. Germline filter positive predictive values and sensitivities. (DOCX 22 kb) Additional file 8: Figure S1. Mutational load predictions with different panel tests for the colon adenocarcinoma subset. Comparison of mutational load predictions using WES or either matched (**a**) or unmatched (**b**) large panel tests (*n* = 300 genes) demonstrates both can reliably predict the mutational load. The *linear regression line* is shown in *black* with *95 % confidence bands* shaded in *grey*. The *identity line* (*dashed*) is shown for comparison. With medium sized panels (*n* = 48 genes), this ability decreases in both the matched and unmatched setting and is not possible with small (n = 15) gene panels. Note that hypermutated tumors were excluded from the regression analysis. (PDF 809 kb) Additional file 9: Figure S2. Mutational load predictions with different panel tests for the lung adenocarcinoma subset. Comparison of mutational load predictions using WES or either matched (**a**) or unmatched (**b**) large panel tests (*n* = 300 genes) demonstrates both can reliably predict the mutational load. The *linear regression line* is shown in *black* with *95 % confidence bands* shaded in *grey*. The *identity line* (*dashed*) is shown for comparison. With medium sized panels (*n* = 48 genes), this ability decreases in both the matched and unmatched setting and is not possible with small (*n* = 15) gene panels. (PDF 848 kb)
