# Supplementary material for: The impact of tumor profiling approaches and genomic data strategies for cancer precision medicine
Source: Genome Med. 2016 Jul 26;8:79. doi: 10.1186/s13073-016-0333-9 (PMC4962446; doi:10.1186/s13073-016-0333-9)
Supplement: Additional file 1: Table S1. — Large panel genes. (DOCX 29 kb) [file 13073_2016_333_MOESM1_ESM.docx]

| ABL1 | BRCA2 | CHEK2 | ERCC4 | FUS | KEAP1 | MYCL1 | PIK3CA | RFWD2 | SRSF2 |
| --- | --- | --- | --- | --- | --- | --- | --- | --- | --- |
| AKT1 | BRD4 | CIITA | ERCC5 | GATA3 | KIT | MYCN | PIK3R1 | RHEB | STAG1 |
| AKT2 | BRIP1 | CREBBP | ESR1 | GATA4 | KRAS | MYD88 | PIM1 | RHPN2 | STAG2 |
| AKT3 | BUB1B | CRKL | ETV1 | GATA6 | LINC00894 | NBN | PMS1 | ROS1 | STAT3 |
| ALK | CADM2 | CRLF2 | ETV4 | GLI1 | LMO1 | NEGR1 | PMS2 | RPL26 | STAT6 |
| ALOX12B | CARD11 | CRTC1 | ETV5 | GLI2 | LMO2 | NF1 | PNRC1 | RUNX1 | STK11 |
| APC | CBL | CRTC2 | ETV6 | GLI3 | LMO3 | NF2 | PRAME | SBDS | SUFU |
| AR | CBLB | CSF1R | EWSR1 | GNA11 | MAP2K1 | NFE2L2 | PRDM1 | SDHA | SUZ12 |
| ARAF | CCND1 | CSF3R | EXT1 | GNAQ | MAP2K4 | NFKBIA | PRF1 | SDHAF2 | SYK |
| ARID1A | CCND2 | CTNNB1 | EXT2 | GNAS | MAP3K1 | NFKBIZ | PRKAR1A | SDHB | TCF3 |
| ARID1B | CCND3 | CUX1 | EZH2 | GNB2L1 | MAPK1 | NKX2-1 | PRKCI | SDHC | TCF7L1 |
| ARID2 | CCNE1 | CYLD | FAM46C | GPC3 | MCL1 | NOTCH1 | PRKCZ | SDHD | TCF7L2 |
| ASXL1 | CD274 | DDB2 | FANCA | GSTM5 | MDM2 | NOTCH2 | PRKDC | SETBP1 | TERC |
| ATM | CD58 | DDR2 | FANCC | H3F3A | MDM4 | NPM1 | PRPF40B | SETD2 | TERT |
| ATRX | CD79B | DEPDC5 | FANCD2 | HNF1A | MECOM | NPRL2 | PRPF8 | SF1 | TET2 |
| AURKA | CDC73 | DICER1 | FANCE | HRAS | MEF2B | NPRL3 | PSMD13 | SF3B1 | TLR4 |
| AURKB | CDH1 | DIS3 | FANCF | ID3 | MEN1 | NRAS | PTCH1 | SH2B3 | TNFAIP3 |
| AXL | CDK1 | DMD | FANCG | IDH1 | MET | NTRK1 | PTEN | SLITRK6 | TP53 |
| B2M | CDK2 | DNMT3A | FAS | IDH2 | MITF | NTRK2 | PTK2 | SMAD2 | TSC1 |
| BAP1 | CDK4 | EED | FBXW7 | IGF1R | MLH1 | NTRK3 | PTPN11 | SMAD4 | TSC2 |
| BCL2 | CDK5 | EGFR | FGFR1 | IKZF1 | MLL | PALB2 | PTPRD | SMARCA4 | U2AF1 |
| BCL2L1 | CDK6 | EP300 | FGFR2 | IKZF3 | MLL2 | PARK2 | QKI | SMARCB1 | VHL |
| BCL2L12 | CDK9 | EPHA3 | FGFR3 | INSIG1 | MPL | PAX5 | RAD21 | SMC1A | WRN |
| BCL6 | CDKN1A | EPHA5 | FGFR4 | JAK2 | MSH2 | PBRM1 | RAF1 | SMC3 | WT1 |
| BCOR | CDKN1B | EPHA7 | FH | JAK3 | MSH6 | PDCD1LG2 | RARA | SMO | XPA |
| BCORL1 | CDKN1C | ERBB2 | FKBP9 | KCNIP1 | MTOR | PDGFRA | RB1 | SOCS1 | XPC |
| BLM | CDKN2A | ERBB3 | FLCN | KDM5C | MUTYH | PDGFRB | RBL2 | SOX2 | XPO1 |
| BMPR1A | CDKN2B | ERBB4 | FLT1 | KDM6A | MYB | PHF6 | RECQL4 | SOX9 | ZNF217 |
| BRAF | CDKN2C | ERCC2 | FLT3 | KDM6B | MYBL1 | PHOX2B | REL | SQSTM1 | ZNF708 |
| BRCA1 | CEBPA | ERCC3 | FLT4 | KDR | MYC | PIK3C2B | RET | SRC | ZRSR2 |

Table S1. Large panel genes.
