# Supplementary material for: The impact of tumor profiling approaches and genomic data strategies for cancer precision medicine
Source: Genome Med. 2016 Jul 26;8:79. doi: 10.1186/s13073-016-0333-9 (PMC4962446; doi:10.1186/s13073-016-0333-9)
Supplement: Additional file 2: Table S2. — Medium panel genes. (DOCX 20 kb) [file 13073_2016_333_MOESM2_ESM.docx]

Table S2. Medium panel genes.

| ABL1 | CSF1R | FGFR3 | JAK2 | NOTCH1 | RET |
| --- | --- | --- | --- | --- | --- |
| AKT1 | CTNNB1 | FLT3 | JAK3 | NPM1 | SMAD4 |
| ALK | EGFR | GNA11 | KDR | NRAS | SMARCB1 |
| APC | ERBB2 | GNAQ | KIT | PDGFRA | SMO |
| ATM | ERBB4 | GNAS | KRAS | PIK3CA | SRC |
| BRAF | FBXW7 | HNF1A | MET | PTEN | STK11 |
| CDH1 | FGFR1 | HRAS | MLH1 | PTPN11 | TP53 |
| CDKN2A | FGFR2 | IDH1 | MPL | RB1 | VHL |
