# Supplementary material for: The impact of tumor profiling approaches and genomic data strategies for cancer precision medicine
Source: Genome Med. 2016 Jul 26;8:79. doi: 10.1186/s13073-016-0333-9 (PMC4962446; doi:10.1186/s13073-016-0333-9)
Supplement: Additional file 3: Table S3. — Small panel genes. (DOCX 19 kb) [file 13073_2016_333_MOESM3_ESM.docx]

Table S3. Small panel genes.

| AKT1 | GNA11 | PDGFRA |
| --- | --- | --- |
| BRAF | GNAQ | PIK3CA |
| EGFR | KIT | RET |
| ERBB2 | KRAS | TP53 |
| FOXL2 | MET | PDGFRA |
