# Supplementary material for: The impact of tumor profiling approaches and genomic data strategies for cancer precision medicine
Source: Genome Med. 2016 Jul 26;8:79. doi: 10.1186/s13073-016-0333-9 (PMC4962446; doi:10.1186/s13073-016-0333-9)
Supplement: Additional file 6: Table S6. — Sample sequencing metrics. (DOCX 27 kb) [file 13073_2016_333_MOESM6_ESM.docx]

Table S6. Sample sequencing metrics.

| Patient | Normal MTC | Tumor MTC |
| --- | --- | --- |
| 100002 | 81.9 | 110.0 |
| 100004 | 130.1 | 153.1 |
| 100007 | 160.1 | 173.1 |
| 100014 | 127.7 | 127.5 |
| 100015 | 237.3 | 168.9 |
| 100019 | 137.9 | 103.0 |
| 1000247 | 128.7 | 153.3 |
| 100027 | 158.0 | 164.8 |
| 100033 | 107.9 | 185.9 |
| 100036 | 116.5 | 142.7 |
| 100045 | 128.9 | 184.3 |
| 100047 | 100.9 | 139.2 |
| 100048 | 140.9 | 191.9 |
| 100052 | 86.3 | 176.4 |
| 100054 | 151.6 | 185.4 |
| 100056 | 152.1 | 140.8 |
| 100060 | 80.8 | 109.5 |
| 100072 | 123.6 | 128.1 |
| 100076 | 166.2 | 140.5 |
| 100082 | 72.6 | 105.3 |
| 100083 | 84.7 | 176.9 |
| 100084 | 84.2 | 176.0 |
| 100086 | 88.9 | 103.5 |
| 100088 | 138.6 | 145.7 |
| 100095 | 127.4 | 159.6 |
| 100101 | 108.5 | 157.6 |
| 100115 | 142.0 | 160.7 |
| 100118 | 131.9 | 236.8 |
| 100121 | 114.1 | 144.2 |
| 100131 | 151.4 | 175.3 |
| 100132 | 135.9 | 158.8 |
| 100138 | 90.7 | 122.4 |
| 100158 | 105.8 | 168.5 |
| 100159 | 116.9 | 161.2 |
| 100169 | 155.6 | 197.6 |
| 100172 | 64.3 | 98.2 |
| 100185 | 74.4 | 118.4 |
| 100188 | 171.3 | 125.2 |
| 100189 | 96.2 | 138.2 |
| 100196 | 149.6 | 189.0 |
| 100199 | 97.2 | 158.7 |
| 100214 | 106.1 | 126.5 |
| 100215 | 112.7 | 162.6 |
| 100232 | 103.7 | 211.3 |
| 100240 | 101.5 | 100.2 |
| 100255 | 90.3 | 118.7 |
| 100263 | 97.9 | 130.0 |
| 100269 | 174.4 | 150.1 |
| 100280 | 64.7 | 88.8 |
| 100281 | 86.3 | 90.8 |
| 100302 | 124.8 | 141.9 |
| 100313 | 117.8 | 219.3 |
| 100317 | 84.5 | 89.0 |
| 100320 | 159.6 | 146.6 |
| 100328 | 183.3 | 212.0 |
| 100334 | 134.7 | 151.0 |
| 100351 | 139.4 | 160.9 |
| 100353 | 189.4 | 195.7 |
| 100370 | 132.1 | 230.0 |
| 100382 | 141.7 | 128.5 |
| 100384 | 181.7 | 162.4 |
| 100387 | 160.7 | 162.1 |
| 100393 | 152.2 | 181.2 |
| 100402 | 166.3 | 202.0 |
| 100414 | 138.1 | 129.1 |
| 100418 | 167.2 | 166.2 |
| 100419 | 101.8 | 94.9 |
| 100425 | 150.2 | 141.0 |
| 100441 | 161.7 | 207.5 |
| 100442 | 195.9 | 224.2 |
| 100452 | 160.4 | 135.0 |
| 100460 | 123.3 | 145.7 |
| 100472 | 173.9 | 171.9 |
| 100489 | 153.4 | 190.3 |
| 100493 | 144.6 | 157.7 |
| 100500 | 130.2 | 191.9 |
| 100508 | 146.8 | 152.1 |
| 100514 | 134.6 | 71.4 |
| 100518 | 126.0 | 167.4 |
| 100562 | 121.0 | 69.8 |
| 100566 | 156.4 | 160.8 |
| 100586 | 135.8 | 214.7 |
| 200005 | 93.4 | 79.8 |
| 200006 | 142.8 | 161.9 |
| 200008 | 145.6 | 199.9 |
| 200009 | 110.9 | 135.6 |
| 200013 | 156.2 | 142.4 |
| 200014 | 81.2 | 166.6 |
| 200016 | 114.7 | 145.0 |
| 200017 | 86.2 | 74.1 |
| 200019 | 159.2 | 250.4 |
| 200022 | 169.5 | 196.9 |
| 200023 | 146.4 | 138.2 |
| 200028 | 164.6 | 173.1 |
| 200031 | 187.1 | 140.2 |
| 200033 | 90.9 | 101.1 |
| 200035 | 75.6 | 99.8 |
| 200036 | 154.6 | 108.1 |
| 200038 | 129.3 | 201.4 |
| 200039 | 174.0 | 178.4 |
| 200042 | 150.3 | 154.4 |
| 200043 | 58.7 | 84.5 |
| 200045 | 115.6 | 129.8 |
| 200054 | 141.9 | 171.7 |
| 200055 | 99.5 | 124.4 |
| 200056 | 80.1 | 102.5 |
| 200057 | 172.2 | 149.3 |
| 200064 | 157.8 | 187.2 |
| 200067 | 129.5 | 191.3 |
| 200068 | 133.8 | 100.7 |
| 200073 | 129.5 | 191.0 |
| 200081 | 98.8 | 104.5 |
| 200083 | 150.1 | 133.3 |
| 200087 | 95.5 | 122.6 |
| 200088 | 98.6 | 110.9 |
| 200089 | 145.3 | 105.7 |
| 200090 | 149.4 | 190.0 |
| 200096 | 158.0 | 138.4 |
| 200097 | 153.3 | 141.0 |
| 200098 | 137.8 | 102.7 |
| 200102 | 65.3 | 103.3 |
| 200107 | 135.6 | 190.0 |
| 200109 | 161.2 | 135.6 |
| 200114 | 187.9 | 194.8 |
| 200121 | 141.8 | 119.0 |
| 200123 | 147.7 | 136.8 |
| 200125 | 204.0 | 155.8 |
| 200127 | 114.6 | 129.6 |
| 200134 | 136.9 | 154.5 |
| 200137 | 154.9 | 144.9 |
| 200140 | 121.7 | 158.7 |
| 200142 | 129.1 | 153.1 |
| 200143 | 130.1 | 158.1 |
| 200144 | 150.3 | 210.2 |
| 200153 | 168.9 | 169.4 |
| 200160 | 151.2 | 226.3 |
| 200162 | 134.2 | 67.5 |
| 200163 | 196.7 | 218.6 |
| 200164 | 194.6 | 218.5 |
| 200168 | 147.9 | 211.7 |
| 200170 | 126.3 | 158.5 |
| 200177 | 189.4 | 149.4 |
| 200180 | 142.7 | 176.3 |
| 200181 | 163.2 | 180.9 |
| 200186 | 106.6 | 172.8 |
| 200187 | 129.5 | 198.8 |
| 200191 | 111.7 | 137.9 |
| 200192 | 106.9 | 239.4 |
| 200193 | 114.6 | 54.3 |
| 200195 | 132.3 | 85.6 |
| 200196 | 129.0 | 83.1 |
| 200197 | 154.8 | 215.3 |
| 200198 | 151.8 | 136.2 |
| 200201 | 162.8 | 223.1 |
| 200205 | 143.7 | 241.9 |
| 200213 | 145.8 | 224.1 |
| 200217 | 159.7 | 168.0 |
