# Supplementary material for: The impact of tumor profiling approaches and genomic data strategies for cancer precision medicine
Source: Genome Med. 2016 Jul 26;8:79. doi: 10.1186/s13073-016-0333-9 (PMC4962446; doi:10.1186/s13073-016-0333-9)
Supplement: Additional file 7: Table S7. — Germline filter positive predictive values and sensitivities. (DOCX 22 kb) [file 13073_2016_333_MOESM7_ESM.docx]

Table S7. Germline filter positive predictive values and sensitivities.

|  | WES | | OncoPanel 300 genes | | Illumina 48 genes | | Illumina 15 genes | |
| --- | --- | --- | --- | --- | --- | --- | --- | --- |
|  | **PPV** | **Sensitivity** | **PPV** | **Sensitivity** | **PPV** | **Sensitivity** | **PPV** | **Sensitivity** |
| dbSNP | 0.369 | 0.922 | 0.578 | 0.726 | 0.756 | 0.613 | 0.879 | 0.671 |
| ExAC - COSMIC (1) | 0.418 | 0.818 | 0.632 | 0.927 | 0.721 | 0.96 | 0.858 | 0.961 |
| ExAC + dbSNP + 1000 Genomes - COSMIC (1) | 0.427 | 0.802 | 0.644 | 0.916 | 0.719 | 0.953 | 0.858 | 0.956 |
| ExAC - COSMIC (3) | 0.494 | 0.766 | 0.722 | 0.862 | 0.796 | 0.887 | 0.865 | 0.908 |
| ExAC - COSMIC (5) | 0.512 | 0.761 | 0.738 | 0.861 | 0.811 | 0.887 | 0.865 | 0.906 |
| ExAC - COSMIC (10) | 0.529 | 0.759 | 0.776 | 0.86 | 0.878 | 0.884 | 0.945 | 0.906 |
| ExAC | 0.559 | 0.755 | 0.778 | 0.784 | 0.871 | 0.77 | 0.946 | 0.83 |
| ExAC + dbSNP + 1000 Genomes | 0.587 | 0.729 | 0.773 | 0.636 | 0.849 | 0.566 | 0.927 | 0.647 |
