# Supplementary figures and images for: The impact of tumor profiling approaches and genomic data strategies for cancer precision medicine
Source: Genome Med. 2016 Jul 26;8:79. doi: 10.1186/s13073-016-0333-9 (PMC4962446; doi:10.1186/s13073-016-0333-9)

Supplementary Figure 1

Colon

A

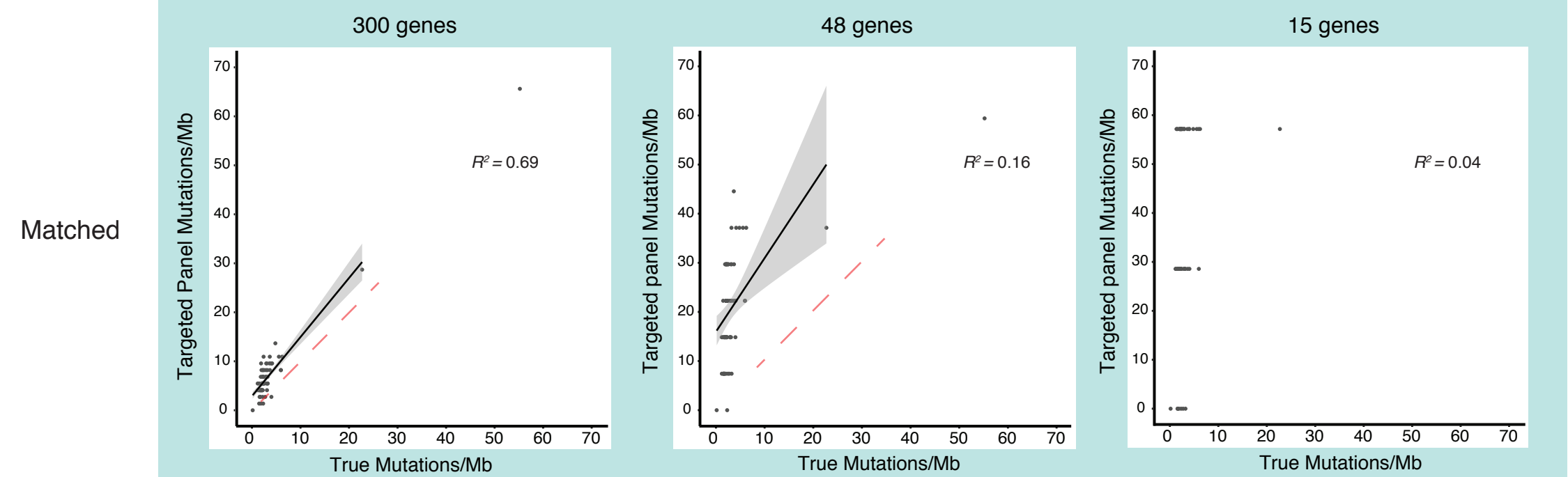

B

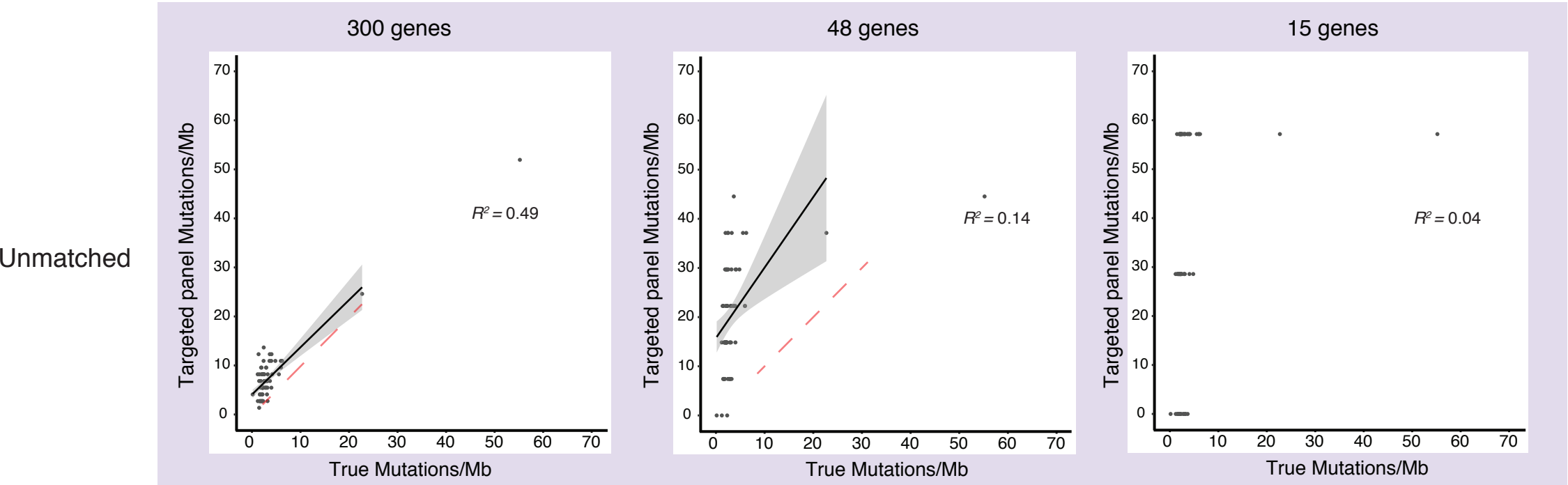

Supplement: Additional file 8: Figure S1. — Mutational load predictions with different panel tests for the colon adenocarcinoma subset. Comparison of mutational load predictions using WES or either matched (a) or unmatched (b) large panel tests (n = 300 genes) demonstrates both can reliably predict the mutational load. The linear regression line is shown in black with 95 % confidence bands shaded in grey. The identity line (dashed) is shown for comparison. With medium sized panels (n = 48 genes), this ability decreases in both the matched and unmatched setting and is not possible with small (n = 15) gene panels. Note that hypermutated tumors were excluded from the regression analysis. (PDF 809 kb) [file 13073_2016_333_MOESM8_ESM.pdf]

Supplementary Figure 2

Lung

A

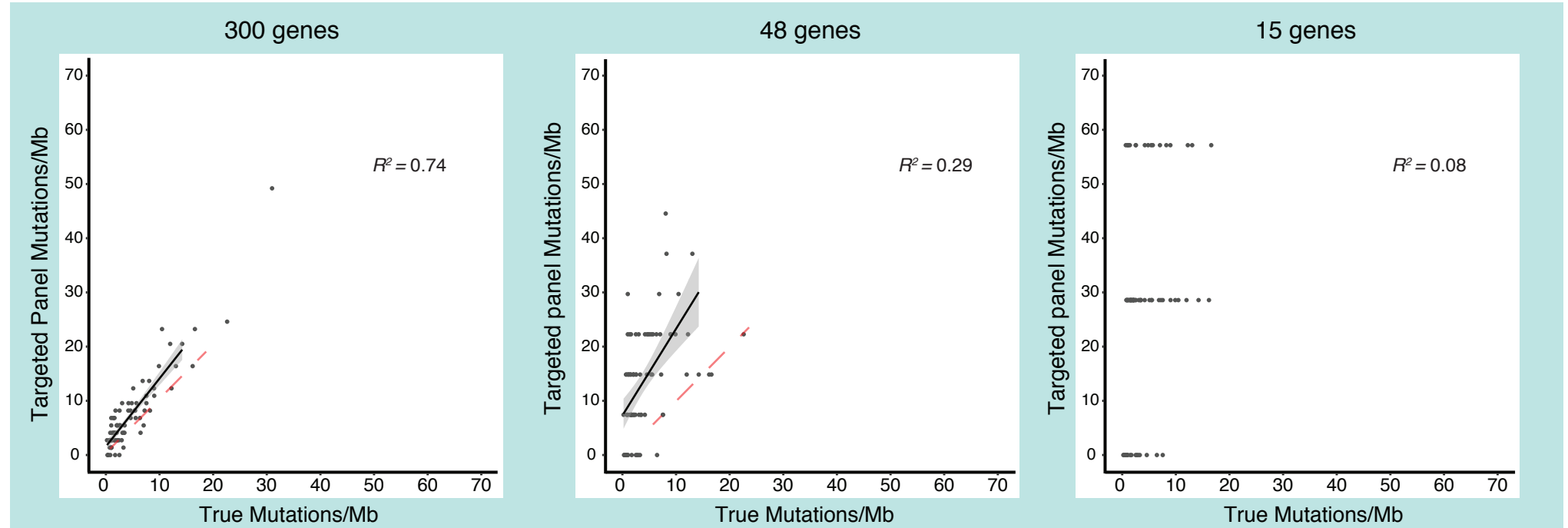

B

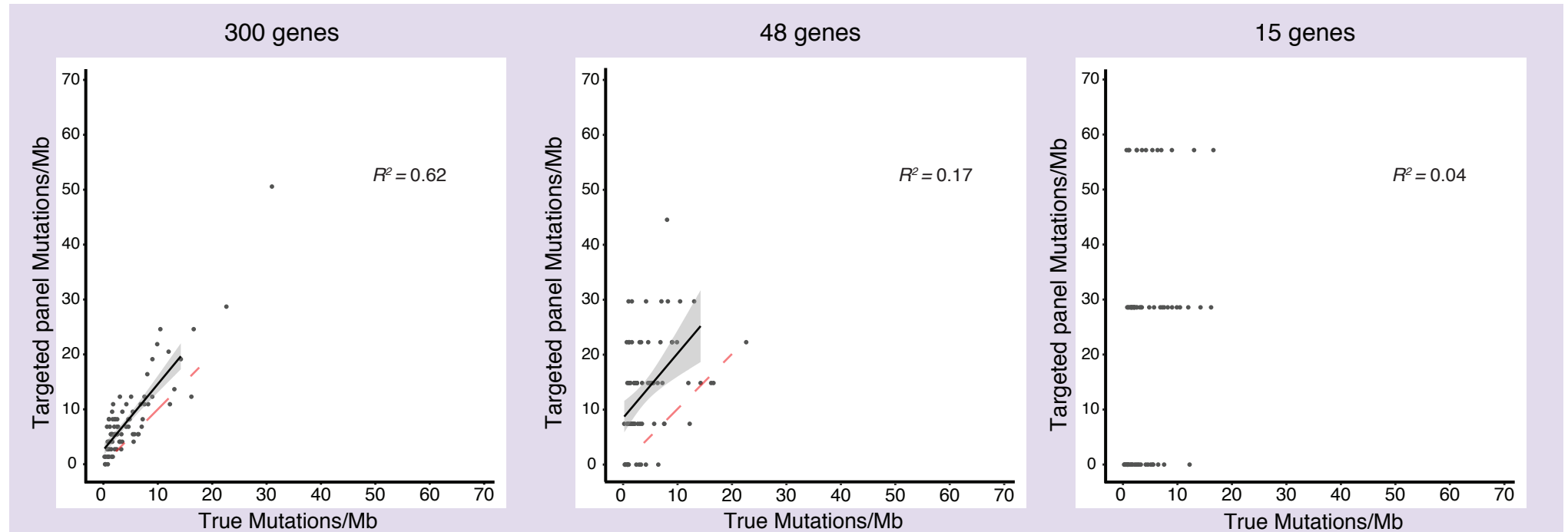

Supplement: Additional file 9: Figure S2. — Mutational load predictions with different panel tests for the lung adenocarcinoma subset. Comparison of mutational load predictions using WES or either matched (a) or unmatched (b) large panel tests (n = 300 genes) demonstrates both can reliably predict the mutational load. The linear regression line is shown in black with 95 % confidence bands shaded in grey. The identity line (dashed) is shown for comparison. With medium sized panels (n = 48 genes), this ability decreases in both the matched and unmatched setting and is not possible with small (n = 15) gene panels. (PDF 848 kb) [file 13073_2016_333_MOESM9_ESM.pdf]
